# *Nrf2* Overexpression in Spontaneously Hypertensive Rats Enhances Adipose Tissue Metabolism through Redox-Mediated Suppression of Mitochondrial Oxidative Phosphorylation

**DOI:** 10.33549/physiolres.935653

**Published:** 2025-12-01

**Authors:** Petr MLEJNEK, Miroslava ŠIMÁKOVÁ, Jan ŠILHAVÝ, Tomáš MRÁČEK, Josef HOUŠTĚK, Irena MARKOVÁ, Martina HÜTTL, Hana MALÍNSKÁ, Michal PRAVENEC

**Affiliations:** 1 Institute of Physiology, Czech Academy of Sciences, Prague, Czech Republic; 2 Center for Experimental Medicine, Institute for Clinical and Experimental Medicine, Prague, Czech Republic

**Keywords:** *Nrf2*, Spontaneously hypertensive rat (SHR), Oxidative stress, Adipose tissue, Metabolism, Mitochondrial function, Oxidative phosphorylation, Antioxidant defense, Insulin sensitivity, Fatty acids, transcriptomics, Transgenic rats, Gene expression

## Abstract

The spontaneously hypertensive rat (SHR) is a widely used model of essential hypertension that also exhibits metabolic disturbances under specific conditions. Oxidative stress plays a central role in the pathogenesis of both hypertension and metabolic dysfunction, with the transcription factor *Nrf2* regulating key antioxidant defenses. Here, we examined whether *Nrf2* overexpression in the SHR improves adipose tissue metabolism. A mouse *Nrf2* transgene under a universal promoter was markedly overexpressed in white adipose tissue, leading to increased insulin sensitivity, reduced saturated fatty acids, and higher n-3 polyunsaturated fatty acids in adipose membrane phospholipids. Transgenic rats also displayed reduced mitochondrial complex I levels, enhanced antioxidant enzyme activities, and decreased lipoperoxidation. Transcriptomic analysis revealed downregulation of oxidative phosphorylation genes. These findings suggest that *Nrf2* overexpression confers antidiabetic and hypolipidemic effects in the SHR, potentially *via* redox-sensitive remodeling of adipose tissue metabolism.

## Introduction

Oxidative stress is a key contributor to the development of insulin resistance, hypertension, and dyslipidemia, hallmarks of the metabolic syndrome [1,2]. The transcription factor *Nrf2* (nuclear factor erythroid 2–related factor 2, encoded by *Nfe2l2* gene) orchestrates cellular antioxidant defense by regulating genes involved in redox homeostasis. In response to oxidative or electrophilic stress, *Nrf2* dissociates from its cytoplasmic repressor *Keap1*, translocates to the nucleus, and activates transcription of antioxidant and detoxification enzymes through the antioxidant response element (ARE) [3].

Beyond redox regulation, *Nrf2* also modulates glucose and lipid metabolism, including pathways such as the pentose phosphate pathway, fatty acid oxidation, and cholesterol homeostasis. In metabolic tissues like fat and liver, *Nrf2* also influences mitochondrial function, lipid composition, and insulin sensitivity [4–7]. However, despite this mechanistic evidence, in vivo studies of the *Nrf2* role in metabolism, especially in rodent models, have produced inconsistent results (reviewed by Li *et al.* [8]). While pharmacological activation of *Nrf2* often improves metabolic outcomes in high-fat diet models [9], genetic overexpression or deletion studies in mice have reported variable phenotypes [10–13].

The spontaneously hypertensive rat (SHR) is a well-established model of essential hypertension and also exhibits metabolic disturbances resembling the human metabolic syndrome [14]. Increased oxidative stress has been implicated in the pathogenesis of spontaneous hypertension and metabolic disturbances in SHR dietary and transgenic models [15–18] or in SHR conplastic strains [19–21], making it a relevant background for exploring redox-driven metabolic adaptations.

In this study, we investigated the effects of *Nrf2* overexpression in a transgenic SHR line. We showed that *Nrf2* upregulation in adipose tissue was associated with improved insulin sensitivity, favorable lipid remodeling, and reduced oxidative damage. These effects were accompanied by downregulation of mitochondrial oxidative phosphorylation genes, suggesting a role for *Nrf2* in metabolic reprogramming of adipose tissue under oxidative stress.

## Materials and Methods

### Animals

Transgenic SHR line (hereafter referred to as the SHR-*Nrf2*) was derived by microinjections of fertilized eggs with the mix of Sleeping Beauty (SB) construct containing mouse *Nrf2* cDNA under control of the SV universal promoter and mRNA of the SB100X transposase as previously reported [22]. We used mouse *Nrf2* transgene in the current study. A mouse *Nrf2* transgene was shown to be functional in transgenic rats because the sequence of *Nrf2* gene and its regulatory mechanisms are highly conserved across mice and rats. For instance, a mouse *Nrf2* expression vector transfected into rat primary cells demonstrated that the mouse *Nrf2* protein was able to induce cytoprotective responses, demonstrating cross-species compatibility [23]. This compatibility is supported by the fact that both mouse and rat *Nrf2* regulate similar sets of antioxidant and detoxification genes, and both species utilize the *Keap1*-*Nrf2*-ARE pathway for cellular protection against oxidative stress.

Genotyping of positive rats was done by PCR with the following primers: m*Nrf2*-413F: 5′-gca act cca gaa gga aca - 3′ and m*Nrf2*-573R: 5′ - agg cat ctt gtt tgg gaa tg - 3′. Insertion site was determined by using transposon display [24]. We studied nonfasted male transgenic rats (N=8) at the age of 4 months compared to age-matched nontransgenic males (N=7). Blood pressure and heart rates were measured in separate groups of rats starting at the age of 2 months (N=10 per group). The rats were housed in an air-conditioned animal facility and allowed free access to standard diet 1320 (Altromin, Lage, Germany) and water. The animal study protocol was approved by the Institutional Ethics Committee of the Institute of Physiology, Czech Academy of Sciences, Prague (protocol code 15-2022-P).

### Nrf2 gene expression determined by real time PCR

Total RNA was extracted from tissues using Trizol reagent (Invitrogen), and cDNA was prepared and analyzed by real-time PCR testing using QuantiTect SYBR Green reagents (Qiagen, Inc.) on an Opticon continuous fluorescence detector (MJ Research). Expression levels of selected genes were normalized relative to the expression of peptidylprolyl isomerase A (*Ppia*) (cyclophilin) gene, which served as an internal control, with results determined in triplicate. We used the following primers: Forward primer: 5′ - act aca gtc cca gca gga ca - 3′, Reverse primer: 5′ - gaa tgt ggg caa cct ggg ag - 3′ (according to Rat NM_001399173.1 and Mouse NM_010902.5 *Nrf2* sequences).

### Parameters of insulin sensitivity in skeletal muscle and adipose tissue

Tissue insulin sensitivity was measured according to insulin-stimulated incorporation of glucose into skeletal muscle glycogen or visceral adipose tissue lipids. Diaphragm or epididymal adipose tissue was incubated for 2 hours in 95 % O_2_ with 5 % CO_2_ in Krebs-Ringer bicarbonate buffer (pH 7.4) containing 0.1 μCi/ml of ^14^C-U glucose, 5 mmol/L of unlabelled glucose and 2.5 mg/ml of bovine serum albumin (Fraction V, Sigma, Czech Republic) with or without 250 μU/ml of insulin. Glycogen and lipids were extracted and incorporation of glucose into glycogen or lipids was determined by scintillation counting.

### Lipolysis in isolated epididymal adipose tissue

For measurement of basal and adrenaline stimulated lipolysis, the distal parts of epididymal adipose tissue were incubated in Krebs-Ringer phosphate buffer containing 3 % bovine serum albumin (Sigma, Fraction V, Czech Republic) at 37 °C, pH 7.4 with or without adrenaline (0.25 μg/ml). The tissues were incubated for 2 hours and the concentrations of NEFA and glycerol in the medium were determined.

### Tissue triglyceride measurements

For determination of triglyceride concentrations in liver, heart and soleus muscle, tissues were powdered under liquid N_2_ and extracted for 16 hours in chloroform:methanol, after which 2 % KH_2_PO_4_ was added and the solution was centrifuged. The organic phase was removed and evaporated under N_2_. The resulting pellet was dissolved in isopropyl alcohol and triglyceride content was determined by enzymatic assay (Erba-Lachema, Brno, Czech Republic).

### Fatty acid profile in epididymal adipose tissue phospholipids

Total lipids were extracted using dichloromethane/methanol (2/1, v/v) according to the Folch method. Individual lipid classes were separated by thin-layer chromatography and converted to fatty acid methyl esters as previously described [25]. Fatty acid methyl esters were separated with gas chromatography using Hewlett-Packard GC system with hydrogen as carrying gas, a flame ionization detector and a carbowax-fused silica capillary column. The profiles of individual fatty acids are reported as the relative percentage of the sum of analyzed fatty acids.

### Biochemical analyses

Serum glucose and triglyceride concentrations were measured by standard enzymatic methods (Erba-Lachema, Brno, Czech Republic). NEFA concentrations were determined with the kit from Roche Diagnostics (Mannheim, Germany). Serum insulin and MCP-1 concentrations were determined using rat ELISA kits (Mercodia, Uppsala, Sweden; eBioscience, USA, respectively).

### Parameters of oxidative stress

The activity of antioxidant enzymes and concentrations of lipoperoxidation products were measured as previously described [15]. Activities of superoxide dismutase (SOD), glutathione peroxidase (GPx), glutathione reductase (GR), and glutathione transferase (GST) were analyzed using Cayman Chemicals assay kits (MI, USA). Concentration of conjugated dienes was determined by extraction in media (heptan:isopropanol 2:1) and measured spectrophoto-metrically in heptan layer. Lipoperoxidation products were assessed based on levels of thiobarbituric acid-reactive substances (TBARS) by assaying the reaction with thiobarbituric acid. Concentrations of reduced form of glutathione (GSH) were determined using HPLC diagnostic kit with fluorescence detection (ChromSystems, Germany).

### Blood pressure and heart rate measurements

Arterial blood pressures were measured continuously by radiotelemetry in paired experiments in conscious, unrestrained males from the SHR-*Nrf2* transgenic and SHR control strains (N=10 per group). All rats were allowed to recover for at least 7 days after surgical implantation of radiotelemetry transducers before the start of blood pressure recordings. Pulsatile pressures were recorded in 5-second bursts every 10 minutes throughout the day and night, and 24-hour averages for systolic, diastolic and mean arterial pressure were calculated for each rat. The results from each rat in the same group were then averaged to obtain the group means.

### Western blotting

Samples of tissue homogenates were denatured at 56 °C for 15 min in a sample lysis buffer (2 % (v/v) 2 mercaptoethanol, 4 % (w/v) SDS, 50 mM Tris HCl, pH 7.0, 10 % (v/v) glycerol, 0.017 % (w/v) Coomassie Brilliant Blue R-250) and Tricine. SDS-PAGE was performed on 10 % (w/v) polyacrylamide slab gels. The gels were blotted onto a PVDF membrane (Immobilon P, Merck Millipore) by semidry electrotransfer at 0.8 mA/cm^2^ for 1 hour. Membranes were blocked in 5 % non-fat dried milk dissolved in Tris buffered saline (TBS; 150 mM NaCl, 10 mM Tris HCl, pH 7.5) for 1 hour at room temperature. Specific primary antibodies were used to assess the content of respiratory chain enzymes (SDHA, a subunit of complex II – ab14715; COX1, an mtDNA-encoded subunit of complex IV – ab14705; F1-α, a subunit of complex V – ab110273, all from Abcam), mitochondrial content (porin – a kind gift from Professor de Pinto) and NRF2 protein. For quantitative detection, the corresponding infra-red fluorescent secondary antibodies (Alexa Fluor 680, Life Technologies; IRDye 800, Rockland Immunochemicals) diluted in TBS supplemented with 0.1 % (v/v) Tween-20 were used. The fluorescence was detected using ODYSSEY infra-red imaging system (LI-COR Biosciences) and the signal was quantified using Aida 3.21 Image Analyzer software.

### Gene expression profiling

Total RNA from epididymal adipose tissue of SHR-*Nrf2* transgenic rats and nontransgenic SHR controls (N=4 per group) was extracted. Quality and concentration of RNA was measured with a NanoDrop 2000 spectrophometer (Thermo Scientific). The RNA integrity was analyzed in Agilent Bioanalyzer 2100. We included only samples with intact RNA profile. Affymetrix GeneChip® Rat Gene 1.0 ST Array System was used for the microarray analysis following the standard protocol [100 ng RNA was amplified with Ambion WT Expression Kit (Applied Biosystems), 5.5 μg single-stranded cDNA was labeled and fragmented with GeneChip WT Terminal Labeling and Hybridization (Affymetrix) and hybridized on the chip according to the manufacturer procedure]. The analysis was performed in three replicates. Data were preprocessed in Partek Genomic Suite (Partek Incorporated). In short, the transcription profiles were background corrected using RMA method, probesets summarized by median polish, quantile normalized and variance stabilized using base-2 logarithmic transformation. Analysis of variance yielded transcripts differentially expressed between analyzed samples (within LIMMA) [26]. Storey’s q values [27] were used to select significant differentially expressed genes (q<0.05). The transcription data are MIAME compliant and deposited in the ArrayExpress database (accession no. E-MTAB-15554).

### Statistical analysis

All data are expressed as means ± S.E.M. Differences between control and experimental groups were evaluated by paired or non-paired t tests as appropriate. Statistical analysis of the gene expression data was performed using the REST XL program that tests for significance by a randomization procedure. The 24 hour mean values of systolic blood pressure were analyzed by repeated measures ANOVA with grouping effect of strain and repeated measurements in time. Statistical significance was defined as P<0.05.

Statistical analyses of gene expression profiles were performed in R and within Bioconductor [28]. Differentially expressed genes were selected for GSEA. We performed GSEA on genes that mapped to KEGG pathways [29] and have defined GO terms (Gene Ontology Consortium, 2000) using the Fisher test and approach of Tian *et al.* [30]. For the purpose of the GSEA, transcripts with nominal P<0.05 were considered differentially expressed [31].

## Results

### Production of SHR-Nrf2 transgenic rats

**Fig. 1:**
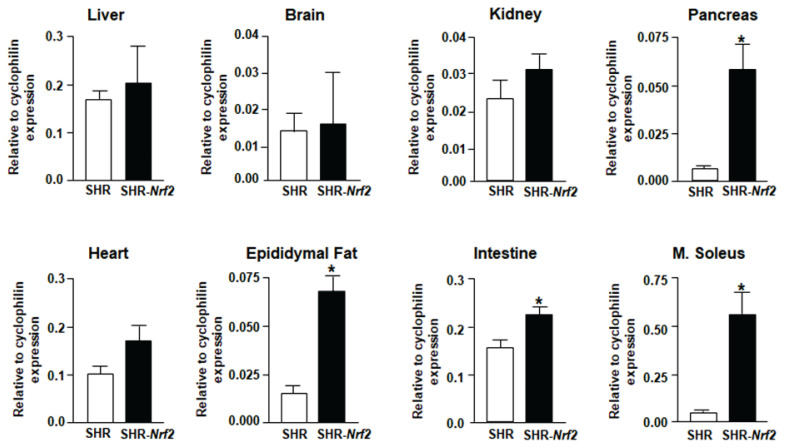
*Nrf2* expression levels in SHR-*Nrf2* transgenic and SHR controls (expression of endogenous *Nrf2* + transgenic *Nrf2* combined). * P<0.05.

A new transgenic line with insertion of the *Nrf2* transgene on chromosome 20p11 outside coding regions was used for experimental testing. Fig. 1 shows increased tissue expression of *Nrf2* gene in transgenic rats. Compared to nontransgenic controls, the most pronounced differences in *Nrf2* expression were observed in pancreas, intestine, fat and soleus muscle while differences in liver, kidney, heart and brain were not statistically significant.

### The effects of transgenic Nrf2 on parameters of oxidative stress and inflammation

As can be seen in Table 1, transgenic expression of *Nrf2* markedly reduced oxidative stress in plasma, liver, heart and kidney. Although concentrations of the intermediate lipoperoxidation products, conjugated dienes, were reduced only in liver, levels of the final lipoperoxidation products, the TBARS, were reduced in plasma, liver, and renal cortex. The activity of antioxidant enzyme SOD was increased only in renal cortex of SHR-*Nrf2* transgenic rats in comparison to SHR controls. The activity of catalase was increased in all investigated tissues. The activities of GSH-dependent enzymes: GSH-Px was activated in plasma, liver and myocardium and GST in liver, myocardium and renal cortex in SHR-*Nrf2* transgenic rats when compared to controls (Table 1). The activity of GSH-regenerating enzyme GR was elevated in liver but concentration of GSH remained unchanged. The unchanged hepatic GSH could be due to the transport of GSH to other tissues which is evidenced by increased plasma GSH concentration. In renal cortex, GR activity and GSH concentration were elevated. Transgenic expression of *Nrf2* was associated with significantly decreased level of pro-inflammatory MCP-1 cytokine (Table 2). Together, these findings support the hypothesis that *Nrf2* overexpression strengthens local redox defenses in insulin-sensitive tissues.

### The effects of transgenic Nrf2 on parameters of glucose and lipid metabolism

**Fig. 2:**
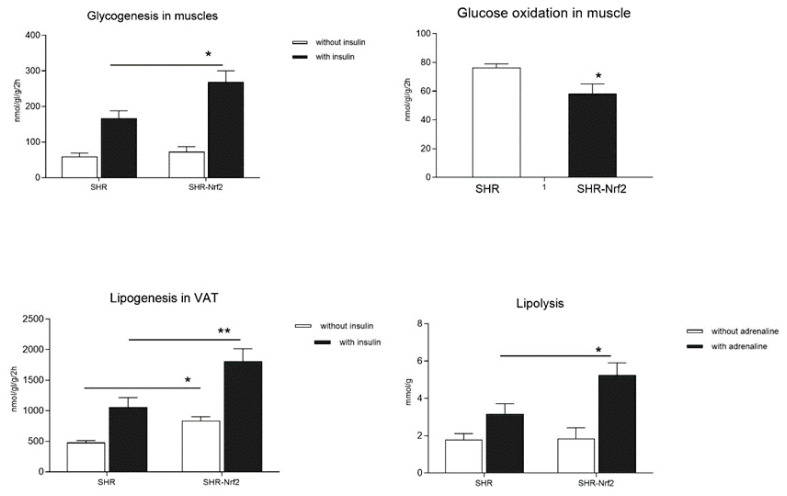
The effect of transgenic expression of *Nrf2* on insulin sensitivity in muscle and adipose tissues, lipolysis and glucose oxidation. **A**) insulin sensitivity of muscle – basal and insulin-stimulated glycogenesis; **B**) glucose oxidation in skeletal muscle; **C**) visceral adipose tissue insulin sensitivity – basal and insulin-stimulated lipogenesis; **D**) basal- and adrenaline-stimulated lipolysis in SHR-*Nrf2* transgenic rats compared to SHR controls. VAT-visceral adipose tissue; * denotes P<0.05; ** denotes P<0.01.

Table 2 shows that transgenic rats exhibited similar levels of plasma glucose but insulin concentrations were significantly reduced when compared to nontransgenic controls which suggests that overexpression of *Nrf2* transgene was associated with increased sensitivity to insulin. This is supported by significantly increased insulin stimulated incorporation of glucose into muscle tissue glycogen (glycogenesis) and adipose tissue lipids (lipogenesis) (Fig. 2). Compared to controls, incremental glycogenesis and lipogenesis were significantly increased in transgenic rats (81±8 vs. 154±8 nmol gl./g/2 h, P<0.00005 and 18±2 vs. 50±6 nmol gl./g/2 h, P=0.0005, respectively). In addition, SHR-*Nrf2* transgenic rats had significantly reduced basal glucose oxidation in muscle tissue (Fig. 2). Transgenic rats exhibited increased plasma NEFA concentrations which might be related to significantly higher adrenaline stimulated lipolysis in adipose tissue when compared to nontransgenic controls (Fig. 2). No significant differences were observed in plasma and liver triglyceride levels while heart and skeletal muscle triglyceride concentrations were significantly reduced in transgenic rats (Table 2).

### The effects of transgenic Nrf2 on fatty acid profile in epididymal adipose tissue phospholipids

**Fig. 3:**
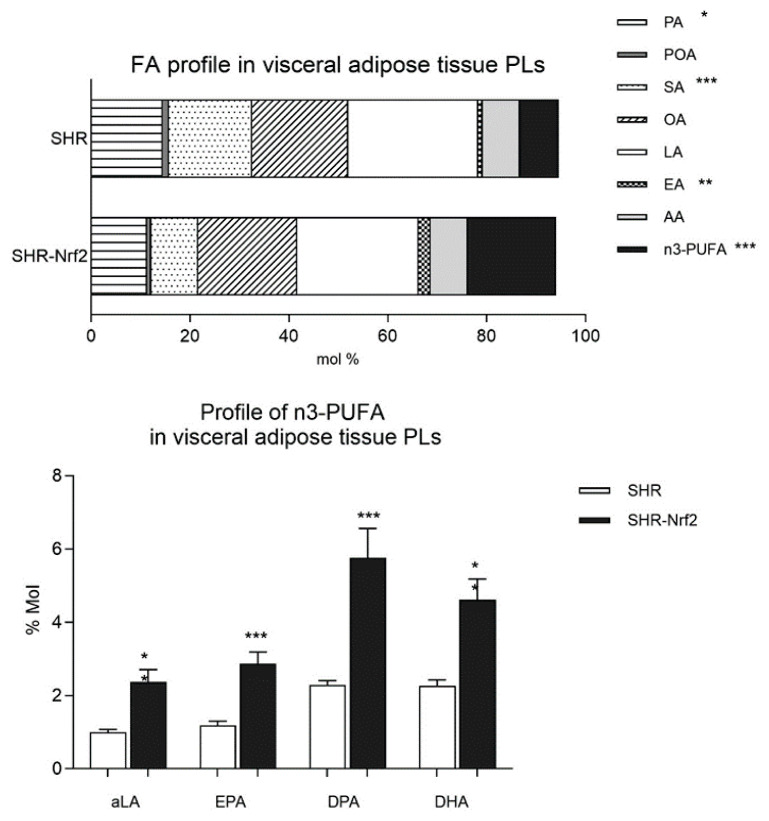
Fatty acid profile in phospholipids in visceral adipose tissue of SHR and SHR-*Nrf2* transgenic animals and fatty acid profile in individual n3-PUFA in visceral adipose tissue phospholipids. FA – fatty acid, PLs – phospholipids, PA – palmitic acid, POA – palmitoleic acid, SA – stearic acid, OA – oleic acid, LA – linoleic acid, EA – eicosadienoic acid, AA – arachidonic acid, aLA – α linoleic acid, EPA – eicosapentaenoic acid, DPA – docosapentaenoic acid, DHA – docosahexaenoic acid. * P<0.05; ** P<0.01, *** P<0.001.

As shown in Fig. 3, transgenic expression of *Nrf2* markedly changed fatty acid (FA) composition in membrane phospholipids in visceral adipose tissue compared to nontransgenic animals. The concentrations of palmitic (16:0) and stearic (18:0) acid were significantly reduced while the concentrations of n3-PUFA, α-linoleic (18:3n3), eicosapentaenoic (20:5n3), docosapentaenoic (22:5n3) and docosahexaenoic acid (22:6n3) were highly significantly elevated. The concentration of n6-PUFA sum was not different between both groups, only the concentration of eicosadienoic acid (20:2n6) was significantly increased in transgenic SHR-*Nrf2* rats (Fig. 3). These changes in FA composition can influence membrane fluidity and signalization and can contribute to the increased insulin sensitivity of visceral adipose tissue and suggest a shift toward a more metabolically favorable lipid profile associated with improved insulin action in transgenic SHR-*Nrf2* rats.

### The effects of transgenic Nrf2 on blood pressure and heart rate

Radiotelemetry blood pressures and heart rates in SHR-*Nrf2* transgenic rats were similar to nontransgenic SHR controls, there were no significant differences (data not shown).

### Mitochondrial OXPHOS enzyme content and mtDNA copy number

**Fig. 4:**
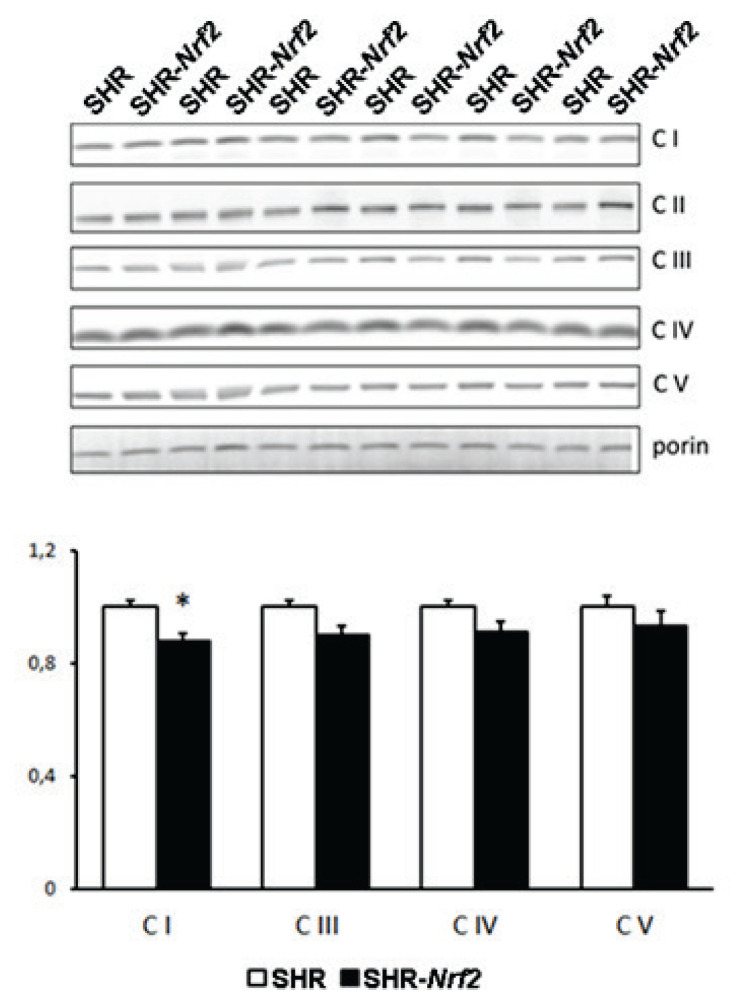
Content of representative OXPHOS complexes subunits in epididymal fat of SHR and SHR-Nrf2 transgenic animals. Quantification is normalized to the content of outer mitochondrial membrane protein porin which serves as a marker of mitochondrial mass. Mitochondrial proteins quantified in homogenates, i.e. data represent total content in tissue. * P<0.05.

Western blot analysis using monoclonal antibodies for selected marker subunits of individual enzyme complexes of mitochondrial respiratory chain in white adipose tissue revealed significant reduction of complex I in SHR-*Nrf2* transgenic rats compared to SHR strain (Fig. 4). Other complexes were also reduced but the differences did not achieve statistical significance. There were no significant differences in mtDNA copy number (data not shown).

To search for molecular mechanisms responsible for reduced oxidative stress, reduced levels of complex I and increased sensitivity to insulin in white adipose tissue, we measured gene expression profiles in epididymal fat. As can be seen in Table 3, increased expression of *Nrf2* was associated with significant downregulation of genes from oxidative phosphorylation, citrate cycle, pyruvate metabolism and steroid synthesis pathways (Table 3).

Together, these results indicate that *Nrf2* overexpression in adipose tissue promoted a coordinated response involving enhanced antioxidant capacity, suppression of mitochondrial oxidative activity, and remodeling of lipid composition. These adaptations were associated with improved insulin sensitivity in the SHR model, linking *Nrf2*-driven redox remodeling to beneficial metabolic outcomes.

## Discussion

In this study, we demonstrated that overexpression of *Nrf2* in the SHR enhanced antioxidant defenses and improved adipose tissue insulin sensitivity. The observed upregulation of antioxidant enzymes and reduction in lipid peroxidation products are consistent with the canonical role of *Nrf2* in enhancing redox defense. These redox changes were paralleled by favorable lipid remodeling, including decreased saturated fatty acids and increased n-3 polyunsaturated fatty acids, lipid shifts that are known to enhance membrane fluidity and insulin signaling [32].

One of the most striking findings was the downregulation of oxidative phosphorylation (OXPHOS) genes and reduced complex I protein levels in adipose tissue. Mitochondrial metabolism is both a source and target of oxidative stress; thus, its suppression may serve as a protective adaptation to minimize reactive oxygen species (ROS) generation under chronic stress [33]. White adipose tissue has relatively low baseline mitochondrial content compared to oxidative tissues like muscle or liver, making it particularly sensitive to shifts in mitochondrial gene expression or function [34,35]. Mechanistically, *Nrf2*-mediated suppression of OXPHOS gene expression in adipose tissue may arise from both direct and indirect effects. Direct repression may occur via recruitment of corepressors by *Nrf2* or interference of *Nrf2* with mitochondrial gene promoters, while indirect mechanisms may involve upregulation of the pentose phosphate pathway and downregulation of mitochondrial biogenesis pathways [4,35–37]. Additionally, *Nrf2* activation can modulate secondary redox-sensitive transcriptional regulators such as HIF-1α and ATF4, both of which have been implicated in OXPHOS repression and metabolic adaptation under stress [8,33]. Future studies should address the potential cross-talk between *Nrf2* and these pathways in adipose tissue.

At the systemic level, the suppression of OXPHOS in adipose tissue may paradoxically contribute to improved insulin sensitivity. By reducing mitochondrial ROS production, *Nrf2* activation could prevent adipose tissue inflammation and lipotoxicity. Additionally, improved adipose tissue insulin sensitivity may reduce ectopic lipid accumulation in peripheral tissues, as evidenced by the decreased triglyceride levels in skeletal and cardiac muscle. Thus, while *Nrf2* activation downregulates mitochondrial energy metabolism in white adipose tissue, this may contribute to a more insulin-sensitive, anti-inflammatory phenotype that protects against systemic metabolic dysfunction.

It is important to note, however, that the effects of *Nrf2* activation or inhibition on energy metabolism and insulin sensitivity can be context-dependent. For example, systemic *Nrf2* knockout in mice has been reported to increase energy expenditure and protect against high-fat diet-induced obesity and insulin resistance in some studies [10,38,39]. On the other hand, deletion of *Nrf2* specifically in adipocytes has been shown to worsen insulin resistance [11,40] which is consistent with our results that demonstrate protective effects of *Nrf2* overexpression in adipose tissue against oxidative stress and metabolic disturbances.

Interestingly, *Nrf2* overexpression had no significant effect on blood pressure or heart rate, suggesting that its beneficial metabolic and anti-inflammatory effects can be uncoupled from cardiovascular regulation in the SHR model. This is consistent with previous studies showing that *Nrf2* activation primarily impacts redox and metabolic pathways, with its influence on hemodynamic parameters being indirect and mediated through improved redox and metabolic homeostasis.

In conclusion, our findings highlight a protective role for *Nrf2* in adipose tissue under conditions of oxidative and metabolic stress. By coordinating antioxidant defense with mitochondrial and lipid remodeling, *Nrf2* improves insulin sensitivity and metabolic balance in the SHR model. These results support the potential of *Nrf2*-targeted strategies for treating components of the metabolic syndrome, particularly in individuals with concurrent hypertension and metabolic dysfunction. Further research is warranted to explore the tissue-specific effects of *Nrf2* activation and to optimize therapeutic approaches targeting the *Nrf2*–mitochondrial axis for metabolic and cardiovascular disease.

## Limitations

A potential limitation of our study is a use of universal promoter to drive *Nrf2* transgene expression, which may result in variable expression patterns across tissues. Although the observed metabolic effects in adipose tissue are robust and consistent with a primary role for *Nrf2* in this tissue, systemic effects from *Nrf2* overexpression in other organs cannot be entirely ruled out. Future studies using tissue-specific promoters will help to further clarify the relative contributions of adipose versus systemic *Nrf2* activation to the observed metabolic improvements.

A further limitation of our constitutive transgenic approach is that *Nrf2* was overexpressed from early development, which may have allowed compensatory mechanisms to arise that would not be present with acute *Nrf2* activation in adulthood. Thus, the metabolic improvements observed might differ qualitatively or quantitatively from those resulting from transient or inducible *Nrf2* activation. Employing inducible or time-controlled *Nrf2* expression systems in future studies could help distinguish between developmental adaptations and acute effects, providing a more precise understanding of the role of *Nrf2* in adipose tissue metabolism and redox regulation.

Another limitation of this study is that only male rats were utilized. Since metabolic and redox-regulatory phenomena can show pronounced sex-specific differences, the effects of *Nrf2* overexpression in female SHR rats remain unknown. Future studies including both sexes will be necessary to determine whether the observed metabolic improvements and gene expression changes are generalizable, or if there are distinctive or additional outcomes in females.

## Figures and Tables

**Table 1:** Parameters of oxidative stress associated with transgenic expression of Nrf2 in rats fed a standard diet

Tissue	SHR	SHR-Nrf2
**Plasma**		

*SOD (U/mg)*	1.764±0.157	1.864±0.105
*CAT (μmol H* *_2_* *O* *_2_* */min/ml)*	1170±21	1245±22*
*GPx (μmol NADPH min/ml)*	271±19	345±8**
*GST (nmol CDNB, min/ml)*	4.50±0.67	6.0±0.66
*GR (μmol NADPH/min/ml)*	80±8	87±6
*GSH (μmol/ml)*	5.53±0.19	6.55±0.18**
*CD (nM/ml)*	32.6±1.1	34.0±1.4
*TBARS (nmol/ml)*	1.701±0.110	1.070±0.101***

**Liver**		

*SOD (U/mg * *_prot_* *)*	0.114±0.017	0.102±0.007
*CAT (μmol H* *_2_* *O* *_2_* */min/mg * *_prot_* *)*	977±25	1251±25***
*GPx (μmol NADPH min/mg * *_prot_* *)*	206±14	288±18**
*GST (nmol CDNB, min/mg * *_prot_* *)*	116±11	162±15*
*GR (μmol NADPH/min/mg * *_prot_* *)*	101±6	142±15*
*GSH (μmol/mg * *_prot_* *)*	39±4	41±3
*CD (nM/mg * *_prot_* *)*	36.0±3.4	27.6±2.1*
*TBARS (nmol/mg * *_prot_* *)*	1.637±0.165	0.957±0.097**

**Myocardium**		

*SOD (U/mg * *_prot_* *)*	0.053±0.007	0.051±0.004
*CAT (μmol H* *_2_* *O* *_2_* */min/mg * *_prot_* *)*	712±49	881±56*
*GPx (μmol NADPH min/mg * *_prot_* *)*	137±10	256±24***
*GST (nmol CDNB, min/mg * *_prot_* *)*	22±3	36±3**
*GR (μmol NADPH/min/mg * *_prot_* *)*	49±5	56±6
*GSH (μmol/mg * *_prot_* *)*	24.9±2.5	27.4±1.7
*CD (nM/mg * *_prot_* *)*	18.5±1.3	21.3±1.7
*TBARS (nmol/mg * *_prot_* *)*	0.591±0.059	0.480±0.029

**Renal cortex**		

*SOD (U/mg * *_prot_* *)*	0.039±0.003	0.060±0.004**
*CAT (μmol H* *_2_* *O* *_2_* */min/mg * *_prot_* *)*	646±50	940±48***
*GPx (μmol NADPH min/mg * *_prot_* *)*	146±10	157±16
*GST (nmol CDNB, min/mg * *_prot_* *)*	48±4	70±7*
*GR (μmol NADPH/min/mg * *_prot_* *)*	25±2	33±3*
*GSH (μmol/mg * *_prot_* *)*	11.7±1.4	15.9±0.9*
*CD (nM/mg * *_prot_* *)*	16.7±2.3	16.6±1.4
*TBARS (nmol/mg * *_prot_* *)*	0.691±0.050	0.552±0.037*

*** p<0.001,

** p<0.01,

* p<0.05

**Table 2:** Parameters of glucose and lipid metabolism in rats fed a standard chow

Trait	SHR	SHR-*Nrf2*
*Body weight (g)*	350±6	361±9
*Relative weight of epididymal fat (g/100 g BW)*	1.07±0.03	1.05±0.02
*Relative weight of liver (g/100 g BW)*	3.25±0.04	3.10±0.04*
*Plasma non-fasting glucose (mmol/L)*	5.0±0.3	4.7±0.2
*Plasma triglycerides (mmol/L)*	0.42±0.04	0.39±0.01
*Plasma NEFA (mmol/L)*	0.31±0.02	0.53±0.03**
*Plasma insulin (nmol/L)*	0.407±0.056	0.231±0.033*
*Plasma MCP-1 (ng/mL)*	8.88±0.69	6.54±0.87*
*Heart triglycerides (μmol/g)*	2.10±0.19	1.41±0.14*
*Liver triglycerides (μmol/g)*	8.10±0.41	8.59±0.34
*Liver cholesterol (μmol/g)*	9.84±0.49	9.91±0.50
*Muscle triglycerides (μmol/g)*	3.75±0.96	2.29±0.42*
*Kidney triglycerides (μmol/g)*	3.29±0.54	3.08±0.36

* P<0.05;

** P<0.01

**Table 3:** KEGG pathways determined by GSEA analysis in white adipose tissue

GSEA on KEGG pathways	FDR (GSEA)	Genes with altered expression (P<0.05)
*Oxidative phosphorylation*	3.3e-23	↓Ndufa11, ↓Ndufb2, ↓Ndufv2, ↓Atp5j, ↓Ndufv3, ↓Cox17, ↓Ndufab1, ↓Atp4b, ↓Sdhd, ↓Atp5a1, ↓Atp6v1e2, ↓Uqcrh, ↓Ndufb7, ↓Ndufb8, ↓Uqcrfs1, ↓Sdhb, ↓Ndufb6, ↓Ndufb5, ↓Uqcrb, ↓Atp5o, ↓Ndufs4, ↓Cox8a, ↓Atp5g3, ↓Cox7b, ↓Ndufs3, ↓Sdha, ↓Atp6v1e1, ↓Uqcrc1, ↓Cyc1, ↓Ndufa6, ↓Ndufc2, ↓Ndufa9, ↓Cox7c, ↓Atp6v0e2, ↓Ndufv1, ↓Ndufa7, ↓Ndufs8, ↓Uqcrc2, ↓Atp5f1, ↓Atp5d, ↓Sdhc, ↓Ndufb10, ↓Ndufa5, ↓Cox5b, ↓Atp6v1a, ↓Ndufb11, ↓Atp5i, ↓Uqcrq, ↓Ndufa2, ↓Cox4i1
*Citrate cycle (TCA cycle)*	7.58e-11	↓Idh3a, ↓Pdhb, ↓Acly, ↓Sdhd, ↓Mdh2, ↓Sdbh, ↓Pdha1, ↓Sdha, ↓Idh3b, ↓Dlat, ↓Dld, ↓Sucla2, ↓Suclg1, ↓Aco2, ↓Fh, ↓Sdhc, ↓Idh2, ↓Pc, ↑Pck1, ↓Cs, ↓Aco1
*Steroid synthesis*	4.84e-06	↓Sc5d, ↓Hsb17b7, ↓Nsdhl, ↓Fdft1, ↓Msmo1, ↓Dhcr24, ↑Faxdc2, ↓Tm7sf2, ↓Dhcr7, ↑Lipa, ↓Ebp
*Pyruvate metabolism*	0.00019	↓Pdhb, ↓Akryb1, ↓Me1, ↓Mdh2, ↓Pdha1, ↓Dlat, ↓Dld, ↓Acss2, ↓Glo1, ↓Acat1, ↓Pc, ↑Acss1, ↑Pck1
*Metabolic patways*	1.88e-06	↑Aldh1a1

↑ and ↓ denote higher and lower expression in SHR-*Nrf2* versus SHR, respectively.
